# How to advance the understanding of multimorbidity in neurodevelopmental disorders using longitudinal research?

**DOI:** 10.1002/jcv2.12147

**Published:** 2023-02-24

**Authors:** Henrik Larsson

**Affiliations:** ^1^ School of Medical Sciences Örebro University Örebro Sweden

**Keywords:** longitudinal research, multimorbidity, neurodevelopmental disorders

Neurodevelopmental disorders (NDDs) are complex, heterogeneous, conditions that show considerable clinical overlap. The concept of NDDs were introduced as an overarching category about 10 years ago in the Diagnostic and Statistical Manual of Mental Disorders, Fifth Edition (DSM‐5) (American Psychiatric Association, 2013). In DSM‐5, the NDDs category includes diagnoses such as Attention‐Deficit/Hyperactivity Disorder (ADHD), Autism, Intellectual Disability (ID), communication disorders, specific learning disorders, and motor disorders (e.g., developmental coordination disorder and tic disorders) (American Psychiatric Association, 2013).

A core characteristic of all NDDs is that they typically onset in childhood. Longitudinal studies with a focus on how NDDs emerge early in life is therefore a very important research area, as it has the potential to help improve early detection and identify new early intervention targets for NDDs. Two of the papers in the March 2023 issue of JCPP Advances present detailed explorations of the developmental antecedents of NDDs. The publication by Rudling et al. (2023) demonstrated that, already at 2 years of age, parental ratings of decontextualized language is associated to clinician‐rated symptoms of autism.

The publication by Christoforou et al. (2023) presents results of a carefully conducted systematic review of studies examining preschool executive functioning in children with and without autism or ADHD. The identified studies suggest that pre‐schoolers with autism or ADHD demonstrate robust impairments in global executive functioning. The included studies also seem to suggest that pre‐schoolers with autism appear to be consistently impaired mainly in shifting, while pre‐schoolers with ADHD are more consistently impaired in inhibition, planning and working memory. These two papers in JCPP Advances have both potential implications for early detection and new interventions, but more research is needed.

The construct validity of NDDs is supported by the high rates of comorbidity between various disorders within this diagnostic grouping (American Psychiatric Association, 2013). Individuals with NDDs are also at a high risk for other co‐occurring mental health problems. For example, over 70% of individuals with ADHD meet the diagnostic criteria for at least one additional psychiatric condition (Faraone et al., 2021).

Two of the papers in the March 2023 issue of JCPP Advances contribute to an advanced understanding of co‐occurring mental health problems in individuals with NDDs.

First, the publication by Wren et al. (2023) focused on the extent to which children with persistent speech disorder presents with elevated levels of social, emotional and behavioral difficulties. They used data from the Avon Longitudinal Study of Parents and Children (ALSPAC) covering information about persistent speech disorder at age 8 from recordings and transcriptions of speech samples. A noteworthy strength of this study was that social, emotional and behavioral difficulties were measured across several time points (i.e., at 10–14 years) and using multiple informants, including parent‐, teacher‐ and child‐reported questionnaires and interviews. The main finding from Wren et al. (2023) suggests that children with persistent speech disorder at age 8 were more likely to show peer problems at age 10–11 years compared with their peers, as reported by teachers and parents, whereas teachers were more likely to report problems with emotionality.

Second, the publication by Wolstencroft et al. (2023) explored the extent to which autism moderates the risk of mental health difficulties and parental psychological distress in children with an intellectual or developmental disability of genetic etiology. The study recruited 1904 participants with a copy number variant or single nucleotide variant (5–19 years) from the UK National Health Service. The results of this study indicated that children with both intellectual/developmental disability and autism were at higher risk of emotional and disruptive behavior disorders compared to those with intellectual/developmental disability only. Further, the severity of associated symptoms was also greater in those with autism and intellectual/developmental disability. Finally, parents of children with intellectual/developmental disability and autism also reported greater psychological distress than those with intellectual/developmental disability alone.

The above papers in JCPP Advances make an important contribution to a more complete picture of mental health problems in NDDs. Describing how specific NDDs independently or in combination with other NDDs (e.g., intellectual/developmental + autism) associates with other mental health problems remains important given that most research this far has focused on the most common types of NDDs (i.e., ADHD and autism). Thus, the two papers above add an important focus on understudied subtypes of NDDs.

## FUTURE DIRECTIONS

In recent times, there has been a growing recognition, in particular in general medicine and primary care, of the need to take multimorbidity into account. Multimorbidity is typically defined as the co‐occurrence of two or more chronic disorders in the same person. According to some definitions of multimorbidity, these should affect multiple systems of the body (Skou et al., 2022). Critically, multimorbidity encompasses both physical and mental health, with an increasing body of research recognizing that psychiatric and physical disorders are strongly associated (Arrondo et al., 2022).

For individuals with NDDs, there is still a lack of research focusing on multimorbidity. A first important task for multimorbidity research in NDDs is to advance the understanding of co‐occurring physical health problems. Emerging evidence indicate that individuals with NDDs are at an increased risk for physical health conditions at various stages of the lifespan, including obesity, asthma, epilepsy, and gastrointestinal diseases (Arrondo et al., 2022), but much more research is needed on this topic.

Another important task for future research is to identify potentially modifiable factors that influence the risk of multimorbidity in NDDs. The underlying mechanisms are most likely complex, but modifiable lifestyle and social factors most probably play a role. Although an increasing number of studies have examined multimorbidity interventions, the evidence is still limited (Skou et al., 2022). The field would therefore benefit from greater investment in multimorbidity research in NDDs.

Longitudinal research using observational designs will be critical to address the knowledge gaps surrounding multimorbidity in NDDs, but the reporting and methodological standards of such studies needs to remain high or increase. Several publications in JCPP Advances have made contributions that aims to improve the standards of observational research. For example, an editorial from Larsson (2022) highlighted a need for improved reporting of confounding and more careful interpretation of observational research in psychiatry. Ozonoff et al. (2023), in the current issue of JCPP Advances, address the problem of selective attrition in longitudinal studies. This is important given that many longitudinal studies of NDDs currently fail to report and adequately consider and analyze attrition. An important take home message from Ozonoff et al. (2023) is that an increasing number of studies using longitudinal cohorts need to report both rates of attrition and analyses comparing retained and not‐retained samples to examine the effects of attrition on variables important for internal validity and generalizability. Other recent studies in JCPP Advances have described how triangulation and genetically‐sensitive research designs (Cheesman et al., 2023; Riglin & Stergiakouli, 2022), such as family‐based designs and mendelian randomization can be used to strengthen causal inference in studies of NDDs.

Until recently, there was a general view that NDDs was limited to childhood, but it is now increasingly recognized that NDDs can be lifelong conditions. Not only that, a lifespan perspective on NDDs has been implemented to an increasing extent both in research and clinic settings. Longitudinal studies of individuals with NDDs have played a major role in changing this perspective. Hopefully, longitudinal studies will play an equally important role in transforming the health care of multimorbidity in NDDs.

## AUTHOR CONTRIBUTIONS

**Henrik Larsson:** Conceptualization; Writing – original draft; Writing – review and editing.

## CONFLICT OF INTEREST STATEMENT

Henrik Larsson reports receiving grants from Shire Pharmaceuticals; personal fees from and serving as a speaker for Shire Pharmaceuticals and Evolan Pharma AB outside the submitted work; and sponsorship for a conference on attention‐deficit/hyperactivity disorder from Shire Pharmaceuticals outside the submitted work. He is Editor‐in‐Chief of JCPP *Advances*.
